# Editorial Notes

**Published:** 1904-10

**Authors:** 


### TEXAS MEDICAL JOURNAL.

AUSTIN, TEXAS.

A MONTHLY JOURNAL OF MEDICINE AND SURGERY.

EDITED AND PUBLISHED BY
F. E. DANIEL, M. D.
Mrs. F. E. DANIEL, Managing Editor.

Published Monthly at Austin, Texas. Subscription price $1.00 a year in advance.
Eastern Representative: John Guy Monihan, St. Paul Building, 220 Broadway New York City.
Official organ of the West Texas Medical Association, the Houston District Me ical Association, the Austin District Medical Society, the Brazos Valley Medic a Association, the Galveston County Medical Society, and several others.

### EDITORIAL NOTES.

YE EDITOR and ye Mascot left Austin Sept. 30th for St. Louis, via International & Great Northern, Texas Pacific, and Iron Mountain. Dr. Daniel goes primarily to attend the International Congress on Tuberculosis (October 3d, 4th and 5th), and with Mrs. D. to incidentally take in the Fair. Readers of the "Red Back" will find so much of interest in this number that they will not miss the usual able editorials, and I know will excuse the omission. They would, if they knew how hot it is about now and how much work has to be done and has been done. *Au revoir.* See you later.

I WISH TO URGE the speedy organization of district medical societies where none has yet been organized. Our Councilors are all zealous, and they must get a move on them by the time the leaves begin to fall, for there is much work to be done. We are pleased to be assured from all over the State that enthusiasm in organization and in society work is still at high pitch. Every respectable doctor in Texas must be enrolled. Sorry for the few who will be out in the cold.

NEW ORLEANS POLYCLINIC.—Eighteenth annual session opens November 7, 1904, and closes may 10, 1905. Physicians will find the Polyclinic an excellent means for posting themselves upon modern progress in all branches of medicine and surgery. The specialties are fully taught, including laboratory and cadaveric work. For further information, address New Orleans Polyclinic, postoffice box 797, New Orleans, La.

---

OUR HANDSOME FRIEND Witten Booth Russ, of San Antonio, President Bexar County Medical Society, was appointed to the long term on the Judicial Council American Medical Association. He is Chairman of the State Medical Association Councilors.

---

WILL REPAIR YOUR ELECTRIC MACHINES.—Static and all electrical medical apparatus put in running order. I am also agent for electrical and X-ray apparatus. Oliver Brush, 710 Colorado Street, Austin, Texas.

---

$2500 to $3000 unopposed practice to purchaser of residence and thirty acres fine land in country village. Small stock drugs, and telephone line included. Price, $2000. Address Wm. M. Morgan, M. D., McMahan, Texas.

